# Ethyl 3-eth­oxy­carbonyl­methyl-7-methyl-5-phenyl-5*H*-thia­zolo[3,2-*a*]pyrimidine-6-carboxyl­ate

**DOI:** 10.1107/S1600536812037828

**Published:** 2012-09-08

**Authors:** H. Nagarajaiah, Noor Shahina Begum

**Affiliations:** a Department of Studies in Chemistry, Bangalore University, Bangalore 560 001, India

## Abstract

In the title compound, C_20_H_22_N_2_O_4_S, the central pyrimidine ring incorporating a chiral C atom is significantly puckered and adopts a slight boat conformation with C atom bearing the phenyl ring and the N atom opposite displaced by 0.367 (2) and 0.107 (2) Å, respectively, from the plane formed by the remaining ring atoms. The benzene ring is positioned axially to the pyrimidine ring, making a dihedral angle of 88.99 (5)°. The thia­zole ring is essentially planar (r.m.s. deviation = 0.0033 Å). In the crystal, pairs of C—H⋯O inter­actions result in centrosymmetric dimers with graph-set motifs *R*_1_^2^(7) and *R*_2_^2^(8). A weak C—H⋯π contact is also observed.

## Related literature

For the therapeutic potential of thia­zolopyrimidine derivatives, see: Zhi *et al.* (2008 ▶). For the synthesis of the title compound, see: Nagarajaiah *et al.* (2012 ▶). For a related structure, see: Nagarajaiah & Begum (2011) ▶. For hydrogen-bond motifs, see: Bernstein *et al.* (1995 ▶). For carbonyl–π interactions, see: Gautrot *et al.* (2006 ▶).
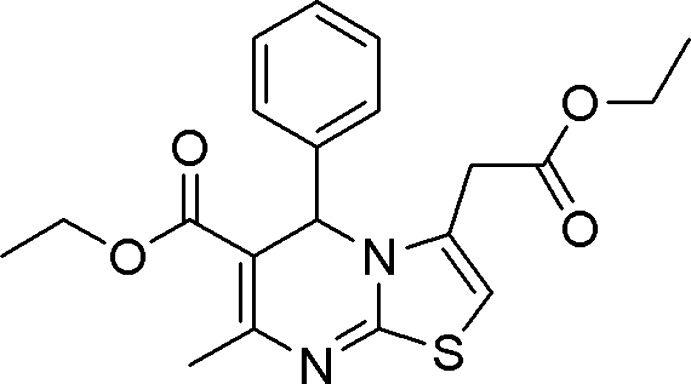


## Experimental

### 

#### Crystal data

C_20_H_22_N_2_O_4_S
*M* *_r_* = 386.46
Monoclinic, 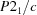

*a* = 10.0861 (4) Å
*b* = 7.7954 (3) Å
*c* = 23.4088 (10) Å
β = 95.000 (3)°
*V* = 1833.52 (13) Å^3^
*Z* = 4
Mo *K*α radiation
μ = 0.21 mm^−1^
*T* = 296 K
0.18 × 0.16 × 0.16 mm

#### Data collection

Bruker SMART APEX CCD detector diffractometer
Absorption correction: multi-scan (*SADABS*; Bruker, 1998 ▶) *T*_min_ = 0.964, *T*_max_ = 0.968
11747 measured reflections
3982 independent reflections
3102 reflections with *I* > 2σ(*I*)
*R*_int_ = 0.037

#### Refinement

*R*[*F* ^2^ > 2σ(*F* ^2^)] = 0.043
*wR*(*F* ^2^) = 0.118
*S* = 1.00
3982 reflections
247 parameters
H-atom parameters constrained
Δρ_max_ = 0.50 e Å^−3^
Δρ_min_ = −0.29 e Å^−3^

### 

Data collection: *SMART* (Bruker, 1998 ▶); cell refinement: *SAINT-Plus* (Bruker, 1998 ▶); data reduction: *SAINT-Plus*; program(s) used to solve structure: *SHELXS97* (Sheldrick, 2008 ▶); program(s) used to refine structure: *SHELXL97* (Sheldrick, 2008 ▶); molecular graphics: *ORTEP-3* (Farrugia, 1997 ▶) and *CAMERON* (Watkin *et al.*, 1996) ▶; software used to prepare material for publication: *WinGX* (Farrugia, 1999 ▶).

## Supplementary Material

Crystal structure: contains datablock(s) global, I. DOI: 10.1107/S1600536812037828/pv2584sup1.cif

Structure factors: contains datablock(s) I. DOI: 10.1107/S1600536812037828/pv2584Isup2.hkl

Supplementary material file. DOI: 10.1107/S1600536812037828/pv2584Isup3.cml

Additional supplementary materials:  crystallographic information; 3D view; checkCIF report

## Figures and Tables

**Table 1:** Hydrogen-bond geometry (Å, °) *Cg* is the centroid of the thia­zolopyrimidine ring.

*D*—H⋯*A*	*D*—H	H⋯*A*	*D*⋯*A*	*D*—H⋯*A*
C17—H17*A*⋯O2^i^	0.97	2.47	3.415 (3)	164
C5—H5⋯O2^i^	0.98	2.59	3.429 (2)	144
C4—H4*C*⋯*Cg*1^ii^	0.96	3.03	3.897 (4)	151

Symmetry codes: (i) 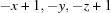; (ii) 
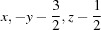
.

**Fig. 1.**
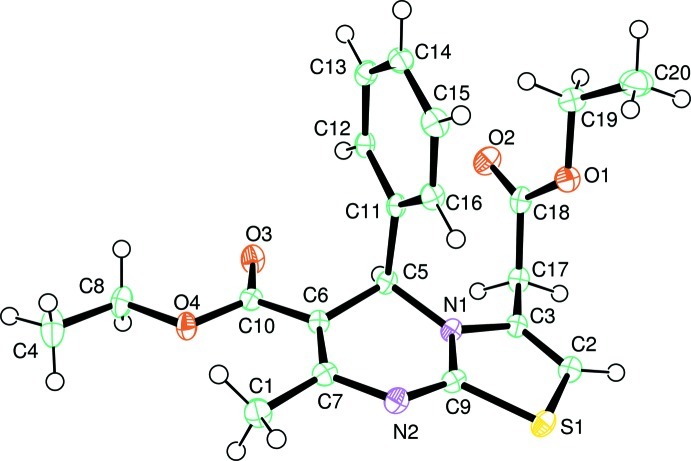
The molecular structure of the title compound with the atom numbering scheme. Displacement ellipsoids are drawn at the 50% probability level. H atoms are presented as small spheres of arbitrary radius.

**Fig. 2.**
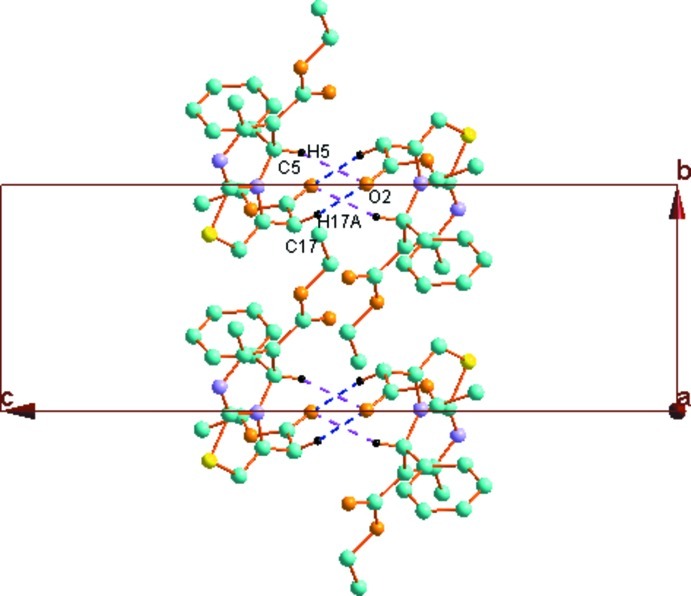
A view of the intermolecular hydrogen bonding interactions (dotted lines) in the crystal structure of the title compound. H atoms non-participating in hydrogen-bonding were omitted for clarity.

## supplementary crystallographic information

### Comment

Thiazolo[3,2-*a*]pyrimidine derivatives may be to generate enzyme inhibitors as novel therapeutical entities for severe neurodegenerative diseases (Zhi *et al.*, 2008). In continuation to our research interests on thiazolo[3,2-*a*]pyrimidine derivatives (Nagarajaiah & Begum, 2011; Nagarajaiah *et al.* 2012), we report the crystal structure of the title compound.

In the title molecule (Fig. 1), the benzene ring is positioned axially and lies almost perpendicular to the pyrimidine ring (N1/N2/C5/C6/C7/C9) with dihedral angle of 88.99 (5)°. The pyrimidine ring substituted with C5 chiral carbon atom is significantly puckered and adopts a slight boat conformation with N2 and C5 atoms displaced by 0.107 (2) and 0.367 (2) Å, respectively, from the plane formed by the remaining ring atoms. The thiazole ring (S1/N1/C2/C3/C9) is essentially planar with r.m.s.d 0.0033 Å for the fitted atoms. The ethyl carboxylate at C6 is almost co–planar with the thiazolopyrimidine ring with a dihedral angle of 13.40 (5)°, where as the other ethyl carboxylate group at C17 is inclined at an angle of 80.57 (4)° with the thiazolopyrimidine ring and is positioned almost parallel to the benzene ring. This is because of intramolecular carbonyl—π interaction of aryl ring with the ethyl carboxylate group (Gautrot *et al.*, 2006). The exocyclic ester at C8 adopts a *cis* orientation with respect to C8═C9 double bond. The N1—C3 bond length (1.403 (2) Å) in the thiazole ring is longer than that of a typical C═N bond but shorter than a C—N single bond, indicating electron delocalization in the ring. The bond distances and angles in the title compound agree very well with the corresponding bond distances and angles reported in a closely related compound (Nagarajaiah & Begum, 2011).

The crystal structure is stabilized by C—H···O intermolecular interactions involving carbonyl O2 atom, resulting in centrosymmetric dimers; the seven and eight membered rings thus resulting from these interaction can be described as *R*^2^_1_(7) and *R*^2^_2_(8) motifs in graph–set notations (Bernstein *et al.*, 1995). In addition π–ring interaction of the type C—H···Cg (*Cg* being the centroid of the thiazolopyrimidine ring) is also observed in the crystal structure (Table 1 and Fig. 2).

### Experimental

The synthesis of the title compound has already been reported (Nagarajaiah *et al.*, 2012). The crystals suitable for X-ray crystallographic analysis were grown from a solution of ethylacetate.

### Refinement

The H atoms were placed at calculated positions in the riding model approximation with C—H = 0.93, 0.96, 0.97 and 0.98 Å for aryl, methyl, methylene and methyne H-atoms, respectively, with *U*_iso_(H) = 1.5*U*_eq_(C) for methyl H atoms and *U*_iso_(H) = 1.2*U*_eq_(C) for other H atoms.

### Crystal data

**Table:**

C_20_H_22_N_2_O_4_S	*F*(000) = 816
*M**_r_* = 386.46	*D*_x_ = 1.400 Mg m^−^^3^
Monoclinic, *P*2_1_/*c*	Mo *K*α radiation, λ = 0.71073 Å
Hall symbol: -P 2ybc	Cell parameters from 3102 reflections
*a* = 10.0861 (4) Å	θ = 1.8–27.0°
*b* = 7.7954 (3) Å	µ = 0.21 mm^−^^1^
*c* = 23.4088 (10) Å	*T* = 296 K
β = 95.000 (3)°	Block, yellow
*V* = 1833.52 (13) Å^3^	0.18 × 0.16 × 0.16 mm
*Z* = 4	

### Data collection

**Table:**

Bruker SMART APEX CCD detector diffractometer	3982 independent reflections
Radiation source: fine-focus sealed tube	3102 reflections with *I* > 2σ(*I*)
Graphite monochromator	*R*_int_ = 0.037
ω scans	θ_max_ = 27.0°, θ_min_ = 1.8°
Absorption correction: multi-scan (*SADABS*; Bruker, 1998)	*h* = −12→12
*T*_min_ = 0.964, *T*_max_ = 0.968	*k* = −6→9
11747 measured reflections	*l* = −29→29

### Refinement

**Table:**

Refinement on *F*^2^	Primary atom site location: structure-invariant direct methods
Least-squares matrix: full	Secondary atom site location: difference Fourier map
*R*[*F*^2^ > 2σ(*F*^2^)] = 0.043	Hydrogen site location: inferred from neighbouring sites
*wR*(*F*^2^) = 0.118	H-atom parameters constrained
*S* = 1.00	*w* = 1/[σ^2^(*F*_o_^2^) + (0.0675*P*)^2^ + 0.5269*P*] where *P* = (*F*_o_^2^ + 2*F*_c_^2^)/3
3982 reflections	(Δ/σ)_max_ < 0.001
247 parameters	Δρ_max_ = 0.50 e Å^−^^3^
0 restraints	Δρ_min_ = −0.29 e Å^−^^3^

### Special details

**Table:**

Geometry. All s.u.'s (except the s.u. in the dihedral angle between two l.s. planes) are estimated using the full covariance matrix. The cell s.u.'s are taken into account individually in the estimation of s.u.'s in distances, angles and torsion angles; correlations between s.u.'s in cell parameters are only used when they are defined by crystal symmetry. An approximate (isotropic) treatment of cell s.u.'s is used for estimating s.u.'s involving l.s. planes.
Refinement. Refinement of *F*^2^ against ALL reflections. The weighted *R*-factor *wR* and goodness of fit *S* are based on *F*^2^, conventional *R*-factors *R* are based on *F*, with *F* set to zero for negative *F*^2^. The threshold expression of *F*^2^ > 2σ(*F*^2^) is used only for calculating *R*-factors(gt) *etc*. and is not relevant to the choice of reflections for refinement. *R*-factors based on *F*^2^ are statistically about twice as large as those based on *F*, and *R*- factors based on ALL data will be even larger.

### Fractional atomic coordinates and isotropic or equivalent isotropic displacement parameters (Å^2^)

**Table:**

	*x*	*y*	*z*	*U*_iso_*/*U*_eq_	
S1	0.18621 (4)	0.21888 (6)	0.308470 (19)	0.01968 (14)	
O1	0.64536 (12)	0.10949 (17)	0.36984 (5)	0.0216 (3)	
O2	0.67845 (13)	0.00457 (18)	0.45941 (6)	0.0260 (3)	
O3	0.31568 (12)	−0.40830 (18)	0.48561 (5)	0.0220 (3)	
O4	0.12682 (12)	−0.51826 (17)	0.44357 (5)	0.0199 (3)	
N1	0.30250 (14)	0.0076 (2)	0.37900 (6)	0.0150 (3)	
N2	0.11268 (14)	−0.1069 (2)	0.32378 (6)	0.0183 (3)	
C1	−0.00093 (18)	−0.3634 (3)	0.34719 (8)	0.0228 (4)	
H1A	0.0277	−0.4809	0.3477	0.034*	
H1B	−0.0437	−0.3361	0.3100	0.034*	
H1C	−0.0625	−0.3465	0.3757	0.034*	
C2	0.32329 (18)	0.2865 (3)	0.35279 (7)	0.0189 (4)	
H2	0.3582	0.3969	0.3524	0.023*	
C3	0.37267 (17)	0.1622 (2)	0.38743 (7)	0.0157 (4)	
C4	0.0304 (2)	−0.7801 (3)	0.47057 (10)	0.0351 (5)	
H4A	−0.0546	−0.7244	0.4652	0.053*	
H4B	0.0287	−0.8646	0.5003	0.053*	
H4C	0.0495	−0.8349	0.4355	0.053*	
C5	0.34164 (16)	−0.1592 (2)	0.40446 (7)	0.0149 (4)	
H5	0.3749	−0.1416	0.4446	0.018*	
C6	0.21811 (16)	−0.2722 (2)	0.40233 (7)	0.0150 (4)	
C7	0.11731 (18)	−0.2487 (2)	0.35981 (7)	0.0177 (4)	
C8	0.1356 (2)	−0.6503 (3)	0.48718 (8)	0.0256 (4)	
H8A	0.1217	−0.6011	0.5242	0.031*	
H8B	0.2228	−0.7035	0.4896	0.031*	
C9	0.19752 (17)	0.0147 (2)	0.33814 (7)	0.0165 (4)	
C10	0.22567 (17)	−0.4028 (2)	0.44727 (7)	0.0167 (4)	
C11	0.44966 (17)	−0.2466 (2)	0.37322 (7)	0.0147 (4)	
C12	0.54419 (17)	−0.3488 (2)	0.40333 (7)	0.0164 (4)	
H12	0.5466	−0.3548	0.4431	0.020*	
C13	0.63526 (17)	−0.4424 (2)	0.37482 (8)	0.0190 (4)	
H13	0.6978	−0.5110	0.3955	0.023*	
C14	0.63328 (18)	−0.4338 (2)	0.31562 (8)	0.0202 (4)	
H14	0.6940	−0.4968	0.2965	0.024*	
C15	0.54006 (18)	−0.3308 (3)	0.28515 (8)	0.0210 (4)	
H15	0.5385	−0.3242	0.2454	0.025*	
C16	0.44914 (18)	−0.2375 (2)	0.31367 (7)	0.0188 (4)	
H16	0.3872	−0.1682	0.2929	0.023*	
C17	0.48437 (17)	0.1764 (2)	0.43372 (7)	0.0181 (4)	
H17A	0.4536	0.1319	0.4689	0.022*	
H17B	0.5041	0.2971	0.4399	0.022*	
C18	0.61252 (18)	0.0854 (2)	0.42323 (7)	0.0190 (4)	
C19	0.76628 (18)	0.0231 (3)	0.35511 (8)	0.0237 (4)	
H19A	0.7534	−0.1002	0.3551	0.028*	
H19B	0.8397	0.0508	0.3831	0.028*	
C20	0.7966 (2)	0.0815 (3)	0.29736 (9)	0.0362 (6)	
H20A	0.7213	0.0601	0.2703	0.054*	
H20B	0.8724	0.0201	0.2859	0.054*	
H20C	0.8156	0.2022	0.2984	0.054*	

### Atomic displacement parameters (Å^2^)

**Table:**

	*U*^11^	*U*^22^	*U*^33^	*U*^12^	*U*^13^	*U*^23^
S1	0.0253 (2)	0.0184 (3)	0.0147 (2)	0.0029 (2)	−0.00124 (17)	0.00322 (18)
O1	0.0244 (6)	0.0245 (8)	0.0158 (6)	0.0014 (6)	0.0020 (5)	0.0011 (5)
O2	0.0318 (7)	0.0281 (8)	0.0173 (7)	0.0032 (6)	−0.0033 (5)	0.0032 (6)
O3	0.0260 (7)	0.0255 (8)	0.0138 (6)	−0.0037 (6)	−0.0028 (5)	0.0060 (5)
O4	0.0233 (6)	0.0185 (7)	0.0180 (6)	−0.0037 (6)	0.0021 (5)	0.0045 (5)
N1	0.0186 (7)	0.0158 (8)	0.0103 (7)	0.0005 (6)	−0.0001 (5)	0.0002 (6)
N2	0.0208 (7)	0.0194 (9)	0.0141 (7)	0.0005 (7)	−0.0015 (6)	0.0011 (6)
C1	0.0219 (9)	0.0263 (11)	0.0194 (9)	−0.0026 (8)	−0.0033 (7)	0.0028 (8)
C2	0.0241 (9)	0.0169 (10)	0.0158 (9)	−0.0006 (8)	0.0018 (7)	−0.0022 (7)
C3	0.0212 (8)	0.0151 (10)	0.0113 (8)	−0.0003 (7)	0.0038 (6)	−0.0020 (7)
C4	0.0472 (13)	0.0245 (12)	0.0348 (12)	−0.0122 (11)	0.0094 (10)	0.0019 (10)
C5	0.0208 (8)	0.0151 (10)	0.0084 (8)	0.0001 (7)	−0.0004 (6)	0.0011 (6)
C6	0.0182 (8)	0.0152 (10)	0.0118 (8)	−0.0012 (7)	0.0021 (6)	−0.0012 (7)
C7	0.0203 (9)	0.0192 (10)	0.0138 (8)	0.0020 (8)	0.0027 (6)	−0.0010 (7)
C8	0.0347 (10)	0.0195 (11)	0.0230 (10)	−0.0028 (9)	0.0043 (8)	0.0070 (8)
C9	0.0209 (8)	0.0192 (10)	0.0094 (8)	0.0038 (8)	0.0011 (6)	0.0005 (7)
C10	0.0198 (8)	0.0172 (10)	0.0133 (8)	0.0008 (8)	0.0035 (7)	−0.0012 (7)
C11	0.0179 (8)	0.0130 (10)	0.0129 (8)	−0.0031 (7)	0.0004 (6)	−0.0002 (7)
C12	0.0209 (8)	0.0159 (10)	0.0122 (8)	−0.0033 (7)	−0.0006 (6)	0.0017 (7)
C13	0.0205 (8)	0.0158 (10)	0.0201 (9)	−0.0012 (8)	−0.0017 (7)	0.0004 (7)
C14	0.0216 (9)	0.0187 (10)	0.0209 (9)	−0.0013 (8)	0.0054 (7)	−0.0043 (8)
C15	0.0254 (9)	0.0265 (11)	0.0112 (8)	−0.0017 (8)	0.0018 (7)	−0.0012 (7)
C16	0.0211 (9)	0.0224 (11)	0.0125 (8)	−0.0010 (8)	−0.0008 (7)	0.0028 (7)
C17	0.0246 (9)	0.0170 (10)	0.0125 (8)	−0.0026 (8)	0.0005 (7)	−0.0010 (7)
C18	0.0259 (9)	0.0160 (10)	0.0146 (9)	−0.0043 (8)	−0.0013 (7)	−0.0017 (7)
C19	0.0230 (9)	0.0238 (11)	0.0243 (10)	0.0029 (8)	0.0013 (7)	−0.0007 (8)
C20	0.0342 (11)	0.0451 (15)	0.0307 (12)	0.0145 (11)	0.0109 (9)	0.0093 (10)

### Geometric parameters (Å, º)

**Table:**

S1—C9	1.7363 (19)	C5—H5	0.9800
S1—C2	1.7367 (19)	C6—C7	1.372 (2)
O1—C18	1.334 (2)	C6—C10	1.461 (2)
O1—C19	1.460 (2)	C8—H8A	0.9700
O2—C18	1.208 (2)	C8—H8B	0.9700
O3—C10	1.220 (2)	C11—C12	1.387 (2)
O4—C10	1.340 (2)	C11—C16	1.395 (2)
O4—C8	1.447 (2)	C12—C13	1.388 (3)
N1—C9	1.365 (2)	C12—H12	0.9300
N1—C3	1.403 (2)	C13—C14	1.386 (3)
N1—C5	1.470 (2)	C13—H13	0.9300
N2—C9	1.302 (2)	C14—C15	1.386 (3)
N2—C7	1.389 (2)	C14—H14	0.9300
C1—C7	1.499 (3)	C15—C16	1.386 (3)
C1—H1A	0.9600	C15—H15	0.9300
C1—H1B	0.9600	C16—H16	0.9300
C1—H1C	0.9600	C17—C18	1.513 (3)
C2—C3	1.332 (3)	C17—H17A	0.9700
C2—H2	0.9300	C17—H17B	0.9700
C3—C17	1.498 (2)	C19—C20	1.483 (3)
C4—C8	1.493 (3)	C19—H19A	0.9700
C4—H4A	0.9600	C19—H19B	0.9700
C4—H4B	0.9600	C20—H20A	0.9600
C4—H4C	0.9600	C20—H20B	0.9600
C5—C6	1.523 (2)	C20—H20C	0.9600
C5—C11	1.525 (2)		
			
C9—S1—C2	91.05 (9)	N2—C9—S1	123.07 (13)
C18—O1—C19	115.91 (14)	N1—C9—S1	109.73 (13)
C10—O4—C8	115.64 (14)	O3—C10—O4	121.68 (16)
C9—N1—C3	114.51 (15)	O3—C10—C6	122.88 (16)
C9—N1—C5	118.99 (15)	O4—C10—C6	115.44 (15)
C3—N1—C5	126.05 (14)	C12—C11—C16	118.65 (16)
C9—N2—C7	115.87 (15)	C12—C11—C5	120.09 (15)
C7—C1—H1A	109.5	C16—C11—C5	121.04 (15)
C7—C1—H1B	109.5	C11—C12—C13	120.78 (16)
H1A—C1—H1B	109.5	C11—C12—H12	119.6
C7—C1—H1C	109.5	C13—C12—H12	119.6
H1A—C1—H1C	109.5	C14—C13—C12	120.20 (17)
H1B—C1—H1C	109.5	C14—C13—H13	119.9
C3—C2—S1	112.24 (15)	C12—C13—H13	119.9
C3—C2—H2	123.9	C13—C14—C15	119.50 (17)
S1—C2—H2	123.9	C13—C14—H14	120.3
C2—C3—N1	112.47 (16)	C15—C14—H14	120.3
C2—C3—C17	127.16 (17)	C14—C15—C16	120.25 (17)
N1—C3—C17	120.27 (16)	C14—C15—H15	119.9
C8—C4—H4A	109.5	C16—C15—H15	119.9
C8—C4—H4B	109.5	C15—C16—C11	120.62 (17)
H4A—C4—H4B	109.5	C15—C16—H16	119.7
C8—C4—H4C	109.5	C11—C16—H16	119.7
H4A—C4—H4C	109.5	C3—C17—C18	116.57 (15)
H4B—C4—H4C	109.5	C3—C17—H17A	108.2
N1—C5—C6	107.96 (13)	C18—C17—H17A	108.2
N1—C5—C11	112.26 (13)	C3—C17—H17B	108.2
C6—C5—C11	110.02 (14)	C18—C17—H17B	108.2
N1—C5—H5	108.8	H17A—C17—H17B	107.3
C6—C5—H5	108.8	O2—C18—O1	124.34 (17)
C11—C5—H5	108.8	O2—C18—C17	123.84 (16)
C7—C6—C10	127.18 (16)	O1—C18—C17	111.80 (15)
C7—C6—C5	119.94 (16)	O1—C19—C20	108.42 (16)
C10—C6—C5	112.84 (14)	O1—C19—H19A	110.0
C6—C7—N2	122.04 (17)	C20—C19—H19A	110.0
C6—C7—C1	126.17 (17)	O1—C19—H19B	110.0
N2—C7—C1	111.77 (15)	C20—C19—H19B	110.0
O4—C8—C4	107.47 (16)	H19A—C19—H19B	108.4
O4—C8—H8A	110.2	C19—C20—H20A	109.5
C4—C8—H8A	110.2	C19—C20—H20B	109.5
O4—C8—H8B	110.2	H20A—C20—H20B	109.5
C4—C8—H8B	110.2	C19—C20—H20C	109.5
H8A—C8—H8B	108.5	H20A—C20—H20C	109.5
N2—C9—N1	127.15 (17)	H20B—C20—H20C	109.5
			
C9—S1—C2—C3	−0.19 (14)	C2—S1—C9—N2	177.08 (15)
S1—C2—C3—N1	0.62 (19)	C2—S1—C9—N1	−0.30 (13)
S1—C2—C3—C17	−175.77 (14)	C8—O4—C10—O3	−1.0 (2)
C9—N1—C3—C2	−0.9 (2)	C8—O4—C10—C6	178.56 (15)
C5—N1—C3—C2	171.25 (15)	C7—C6—C10—O3	−175.52 (17)
C9—N1—C3—C17	175.79 (15)	C5—C6—C10—O3	6.9 (2)
C5—N1—C3—C17	−12.1 (2)	C7—C6—C10—O4	4.9 (3)
C9—N1—C5—C6	−28.50 (19)	C5—C6—C10—O4	−172.68 (14)
C3—N1—C5—C6	159.69 (15)	N1—C5—C11—C12	147.17 (16)
C9—N1—C5—C11	92.94 (17)	C6—C5—C11—C12	−92.58 (19)
C3—N1—C5—C11	−78.87 (19)	N1—C5—C11—C16	−38.2 (2)
N1—C5—C6—C7	28.9 (2)	C6—C5—C11—C16	82.0 (2)
C11—C5—C6—C7	−93.96 (19)	C16—C11—C12—C13	−1.0 (3)
N1—C5—C6—C10	−153.39 (14)	C5—C11—C12—C13	173.77 (16)
C11—C5—C6—C10	83.79 (17)	C11—C12—C13—C14	0.4 (3)
C10—C6—C7—N2	171.60 (16)	C12—C13—C14—C15	0.2 (3)
C5—C6—C7—N2	−11.0 (3)	C13—C14—C15—C16	−0.3 (3)
C10—C6—C7—C1	−6.2 (3)	C14—C15—C16—C11	−0.3 (3)
C5—C6—C7—C1	171.16 (16)	C12—C11—C16—C15	0.9 (3)
C9—N2—C7—C6	−10.1 (2)	C5—C11—C16—C15	−173.75 (17)
C9—N2—C7—C1	167.97 (15)	C2—C3—C17—C18	−110.7 (2)
C10—O4—C8—C4	−171.20 (16)	N1—C3—C17—C18	73.2 (2)
C7—N2—C9—N1	10.8 (3)	C19—O1—C18—O2	3.2 (3)
C7—N2—C9—S1	−166.12 (13)	C19—O1—C18—C17	−178.60 (15)
C3—N1—C9—N2	−176.53 (16)	C3—C17—C18—O2	−138.12 (19)
C5—N1—C9—N2	10.7 (2)	C3—C17—C18—O1	43.6 (2)
C3—N1—C9—S1	0.71 (17)	C18—O1—C19—C20	−172.85 (17)
C5—N1—C9—S1	−172.02 (11)		

### Hydrogen-bond geometry (Å, º)

*Cg* is the centroid of the thiazolopyrimidine ring.

**Table:**

*D*—H···*A*	*D*—H	H···*A*	*D*···*A*	*D*—H···*A*
C17—H17*A*···O2^i^	0.97	2.47	3.415 (3)	164
C5—H5···O2^i^	0.98	2.59	3.429 (2)	144
C20—H20*B*···N2^ii^	0.96	2.70	3.516 (3)	144
C4—H4*C*···*Cg*1^iii^	0.96	3.03	3.897 (4)	151

Symmetry codes: (i) −*x*+1, −*y*, −*z*+1; (ii) *x*+1, *y*, *z*; (iii) *x*, −*y*−3/2, *z*−1/2.
